# Croatian training model for medical teachers

**DOI:** 10.3325/cmj.2013.54.585

**Published:** 2013-12

**Authors:** Neda Pjevač, Gordana Pavleković, Mladenka Vrcić-Keglević, Martina Lovrić-Benčić, Anton Šmalcelj, Tomislav Luetić

**Affiliations:** 1 Department of Educational Technology, Andrija Štampar School of Public Health, University of Zagreb School of Medicine, Zagreb, Croatia; 2 Department of Family Medicine, Andrija Štampar School of Public Health, University of Zagreb School of Medicine, Zagreb, Croatia; 3 Department of Internal Medicine, University Hospital Center Zagreb, University of Zagreb School of Medicine, Zagreb, Croatia; 4 Department of Surgery, University Hospital Center Zagreb, University of Zagreb School of Medicine, Zagreb, Croatia; 5 Croatian Association for Medical Education, Zagreb, Croatia

Medical teaching is a demanding and complex task, and the tendency toward its “professionalization” has been recognized by both academic and professional societies (1). Medical teachers are no longer the resources of knowledge, but guides, role models, collaborators, facilitators, advisers, resource developers, and planners. They are well prepared for their clinical roles but not all of them have been trained for their teaching roles. Therefore, staff/faculty development has become an increasingly important component of medical education and the majority of programs focus on the medical teacher (2). Several models of medical teachers' training are widely available, such as learning from self-experience, peer learning, learning from mistakes and successes, shorter or longer courses or workshops, fellowships, integrated longitudinal programs, and postgraduate courses lasting one or two years (3,4).

## “The Art of Medical Education:” the story so far

Croatia has a long tradition of high-quality medical education. All four medical schools (Zagreb, Rijeka, Split, and Osijek) implemented a new curriculum based on core competences for doctors in the 21st century, introduced and developed innovative teaching and learning methods, training programs for the teaching staff, and quality assurance of the provided training. In 1987, University of Zagreb School of Medicine established the Department of Educational Technology, with the primary task of developing high-quality medical education. Since 1990, this Department has developed and implemented training programs for medical teachers. Short three-day thematic workshops for experienced teachers have been organized several times per year with topics such as specificities of graduate and postgraduate education, continuing professional development, learning from experience, cognitive sciences and medical education, creativity in medical education, writing textbooks and handbooks, Objective Structured Clinical Exam, portfolio, assessment methods, etc. All these activities and permanent professional interests of medical teachers from all Croatian medical schools led to the founding of the Croatian Association for Medical Education (CAME). Since 2000, CAME in collaboration with the Department of Educational Technology has organized one-week courses for junior teachers entitled “The Art of Medical Education.” From 2000 to 2012, the course was attended by 455 participants, mostly from Croatian schools of medicine but also from other biomedical and health sciences faculties (dental medicine, pharmacy, nursing, veterinary medicine).

## Main characteristics of the Croatian training model for medical teachers

“The Art of Medical Education” is a one-week course for junior teachers concentrated on the basics of medical education, enabling teachers early in their career to put into practice contemporary approaches to teaching and learning of medicine. The main task of the course is to provide the following:

• basic understanding of the concepts of medical study, challenges, and dilemmas in teaching and learning;

• theoretical framework for understanding factors influencing the quality of teaching-learning process;

• range of evidence-based strategies, both traditional and innovative methods;

• framework for planning, implementing, and evaluating medical education;

• awareness of ethical issues related to medical education.

Course aims and content are based on the list of teachers’ attitudes, knowledge, and skills recommended by medical educators (5), and oriented not only toward acquiring “practical skills” but also toward promotion of academic culture in medical education.

The course employs multiple instructional methods: self-directed and task-based learning, small group discussions, individual and group projects, microteaching using interactive videos, self-reflection and peer reflection, role-play, demonstration, round-table discussions, and others (Table 1) (6).

**Table 1:** Working format and main contents of the course “The Art of Medical Education”

Day 1	Introduction to the Course. Expectations Pre-test (multiple choice questions) Problem-based learning, problem solving Reforms of Medical Schools, strategies and effectiveness of medical education Panel discussion: Europe and the world – Challenges in medical education
Day 2	Self-directed study (readings, tasks related to the daily content) Curriculum planning and module development: developing learning outcomes Teaching and learning tools: Module planning and implementation Panel discussion: Student as a partner in teaching-learning process
Day 3	Self-directed study (readings, tasks related to the daily content) Educational tools in basic, clinical, and public health sciences Bedside teaching Panel discussion: Patient safety and patient as a partner in teaching-learning process
Day 4	Self-directed study (readings, tasks related to the daily content) Traditional vs innovative methods in medical education Lecture delivering (using videos for self and peer feed-back) Evaluation
Day 5	Self-directed study (readings, tasks related to the daily content) Teaching aids (video and computerized technologies, handouts, textbooks, simulators, skill laboratory, e-learning) Principles and methods in assessment Panel: Assessment and exams as a part of teaching-learning process
Day 6	Final test (Multiple choice questions) Staff development and academic standards Panel: Strategies and policies of high education. Perspectives of Croatian Medical Schools development Course evaluation and future plans

Although the course is mainly intended for younger teachers and is one of the requirements for academic advancement (compulsory for future assistant professors), the mean age of participants was 45. However, this is an age-group that still has enough time to improve their quality of teaching. Participants have various professional backgrounds: basic and preclinical science, clinical, public health, and primary care field, as well as other biomedical fields. Inter-professional cooperation and mutual understanding have been established as an instructional strategy. To obtain a certificate it is not sufficient to simply complete the course: the participants have to prepare an educational module in a written form, present it orally, and discuss in front of a three-member committee, but also preferably in front of teachers from their own department.

The course lasts for six days with ten-hour sessions. The teachers are not experts certificated in pedagogy and didactics, but medical teachers and clinical practitioners with long experience in medical education, positively assessed by students and peers, and very active in collaboration with educational centers and medical education associations in Europe.

Self- and peer-evaluation is carried out on several occasions during the course, while process evaluation is done at the end of the week using quantitative and qualitative methods (written questionnaire and group discussion). The elements that received the highest marks were:

• building on and expanding teachers’ previous teaching and learning experiences,

• directing interests to teaching,

• stimulating experience exchange with colleagues from different departments,

• peer discussion as a continuous support for advancement in teaching,

• satisfaction in communication and sharing doubts with experienced teachers, and

• panel discussions with guests and an overview of the present situation in medical education.

Two aspects received lower marks:

• participation of “older teachers” and key decision makers within the faculty, and

• practical applicability of the skills mastered in the course.

The course’s impact on the quality of teaching was evaluated by teachers’ self-assessment and students’ assessment of the teachers. Teachers reported changes in the post-course period (for example: introduction of innovative methods in their teaching modules, changes in attitudes toward students and medical education, introduction of a small elective course, active engagement within teaching department and medical school activities, interest in medical education research). The greatest change was observed in the development of positive attitudes toward teaching and relationship with students, while active engagement within teaching department and interest in medical education research had not changed much. Students used anonymous questionnaires to compare individual teachers before and after the course and compare them with other teachers at the same department who had not participated in the course.

## Lessons learned

Compared to other “training for trainers” courses in medical education with similar aims (2,3), the Croatian model has at least two strengths:

1. Although the trainers are not pedagogy or didactics experts, they are well experienced medical doctors/teachers sharing their own experiences and reflections with participants.

2. A mixed group of participants (with different prior knowledge, fields of work, interests, expectations, and teaching environment) has a positive impact on participants’ motivation and encouragement, since it is important to establish inter-professional cooperation and mutual understanding as an instructional strategy.

However, at least two dilemmas remain:

1. Do we need (formally) certificated medical teachers or those who are highly internally motivated to invest in their own development? Compulsory training for junior teachers could decrease their motivation (formal attendance and certification), without real improvement in future practice. “Medical teaching is not a private business” (7), therefore all teachers should have an opportunity to take part in it. However, teachers with long teaching experience without theoretical knowledge (associate or full professors) would require different types of programs. A prerequisite for a higher-quality medical education is the introduction of teaching standards as well as licensing and re-licensing of all medical teachers.

2. Do we need a competitive medical teacher or a supportive institutional environment? Successful teaching depends not only on “good teachers” but also on supportive social and teaching environment and opportunities. For example, medical teachers working in hospitals may not have time for teaching. Therefore, responsibility for the quality medical education lies not only on universities and medical teachers but also on the interaction between academic disciplines and health care (7,8).

Based on our experiences with the Croatian training program “The Art of Medical Education” since 2000, we believe that it is important to have a comprehensive model based on deeper understanding of medical education rather than only teach basic pedagogical skills. Training models should be designed according to specific medical school’s needs and context, but always aiming to create an educational climate that encourages and rewards educational leadership, innovation, and excellence. Several authors reported similar challenges in training programs for medical teachers (9,10), but further research on the quality of medical teaching is required to answer the questions posed in this text.
